# Motion-based equilibrium reprocessing therapy a novel treatment method for chronic peripheral vestibulopathies: A pilot study

**DOI:** 10.1097/MD.0000000000007128

**Published:** 2017-06-16

**Authors:** Mirke S. Hondebrink, Agali Mert, Roos van der Lint, J. Alexander de Ru, Peter van der Wurff

**Affiliations:** a Military Rehabilitation Centre Aardenburg, Doorn; b Ciran Rehabilitation Centers, Venlo; c Department of Otolaryngology, Central Military Hospital, Utrecht, The Netherlands.

**Keywords:** desensitization, dizziness, rehabilitation, vestibulopathy

## Abstract

Rehabilitation for vestibular disease is a safe method to partially alleviate symptoms of vertigo. It was hypothesized that principles of military aviation vestibular desensitization procedures that have a success rate of more than 80% can be extrapolated to chronic vestibular disease as well.

The virtual reality motion base computer-assisted rehabilitation environment was used as treatment modality in 17 patients. They were exposed to sinusoidal vertical passive whole body motion in increasing intensity for a maximum of 12 sessions. The Dizziness Handicap Inventory (DHI) was used for assessment of the subjective complaints of vertigo.

The median DHI scores of 50 points at baseline dropped to 22 points (*P* <.001) at follow-up. *Post hoc* analysis showed significant differences in outcome between measurements at baseline and at the end of the treatment, between baseline and follow-up, but not between end of treatment and follow-up.

This pilot study concerning motion-based equilibrium reprocessing therapy (MERT) shows that it is a simple, quick, and well-tolerated treatment option to alleviate symptoms in patients with chronic peripheral vestibulopathies.

## 1 Introduction

Diseases of the vestibular system, vestibulopathies, can present in the acute phase with nystagmus, dizziness, nausea, and vertigo.^[1]^ In general, these are temporary, despite the debilitating initial symptoms. In case symptoms persist, they are usually less severe, but nonetheless can cause social isolatio, and reduce the level of daily activities and participation.^[2]^

Treatment of vestibular dysfunction with drugs, for example, beta-histine, oral corticosteroids, or carbamazepine, is limited in indication and success rate.^[3]^ Compliance is often less than optimal. Patients with chronic dizziness due to vestibular dysfunction are advised to receive counselling and rehabilitation.^[4]^ The current standard noninvasive treatment is the administration of vestibular rehabilitation exercises: patients have to incite their symptoms by performing provocative head movements, thereby, in time, decreasing their symptoms.

The purpose of vestibular rehabilitation is to improve the ability of the central nervous system to compensate for lesions or dysfunction in the sensory integration of vestibular, visual, and somatosensory signals (i.e., proprioceptive afferents and cutaneous afferents). The idea is that by provoking symptoms like dizziness through head movements, head positions, and/or whole body movements, central compensatory mechanisms will occur leading to reduction of symptoms.^[5]^ The route through which these processes lead to symptom reduction is not fully elucidated, partly because the process is also dependent on the underlying lesion, but they involve central nervous reorganization. Main learning principles include adaptation through sensory information and/or behavioral substitution (i.e., reweighing other sensory information) and habituation, a process where a provocative stimulus is administered until it vanishes. Vestibular rehabilitation programs, comprising among others Cawthorne & Cooksey exercises, have varying success rates. Still, these programs are the treatment of choice when central compensation for the symptoms is suboptimal.^[6,7]^ But what to do with patients who do not respond well to a vestibular rehabilitation program and continue to have a lower level of functioning than desired?

Motion sickness in military aviation, which can be seen as an occupational dysfunction of the vestibular system, is treated with desensitization programs, because medication often has operational restrictions. The mainstay of these programs is psycho-education of the vestibular system and a gradually increasing exposure to motion sickness inducing stimuli.^[8–10]^ The Dutch motion sickness desensitization program started in the 1980s has a success rate of more than 80% and is comparable with other Air force motion-sickness desensitization programs.^[8,9]^

Although differences between motion sickness and vestibulopathies exist, there is also an intriguing similarity as physical symptoms in both conditions arise because of a mismatch in sensory integration of vestibular, visual, and somatosensory signals. Patients with peripheral vestibular disease have a normal functioning central nervous system and one might therefore wonder whether a similar, yet less provocative, desensitization program might be appropriate and successful for peripheral vestibulopathies in the chronic phase.

Introducing motion conflicts and hence inducing (slowly increasing) motion sickness, analogous with military desensitization programs, could theoretically be a successful method of treatment. The proof of concept of this approach seems promising and at the Military Rehabilitation Centre Aardenburg, Doorn, The Netherlands (MRC), this novel vestibular rehabilitation program, that we called motion-based equilibrium reprocessing therapy (MERT), has been implemented.^[11]^ The aim of the current pilot study was to evaluate the first results of this program.

## 2 Materials and methods

### 2.1 Study setting

In this retrospective pilot study, we included male and female patients with peripheral vestibulopathies. All patients were referred to the MRC for treatment from Otolaryngology or Neurology departments of several hospitals in the Netherlands.

### 2.2 Patients

We evaluated patients who were referred with dizziness and participated in the desensitization protocol of the MRC to reduce their complaints. Files were identified from the digital database of the MRC using ICD-9 code 386.12, in the period between January 1, 2011 and September 1, 2015. Patient files were assessed for clinical history, examination, diagnosis, and previous treatment. The primary inclusion criterion was a diagnosis of peripheral vestibular dysfunction including benign paroxysmal positioning vertigo (BPPV), Ménière disease ,and vestibular neuritis, with symptoms present for at least 4 months. Peripheral vestibular dysfunction was based on referral diagnosis and letters from the Otolaryngology or Neurology departments. Criterion for exclusion in this study was patients with a primary diagnosis other than a peripheral vestibular deficit explaining their dizziness (i.e., systemic illness, psychiatric disorders, central vestibular disorders, and other neurological disorders). Patients were also excluded if they received other treatment than MERT for their dizziness during the program and if they were unable to fill out the questionnaires. Furthermore, the patient's ages ranged from 18 to 80 year old. Patients signed an informed consent to participate in the program.

### 2.3 Measurement

This is an observational retrospective study. In accordance with our daily practice protocol, patients were asked to fill in the Dizziness Handicap Inventory (DHI) before treatment started (baseline) immediately after the treatment procedure (end) and at regular follow-up appointment (about 3 months after the end of treatment). The DHI is a 25-item self-reported questionnaire designed to quantify how an individual's self-perceived handicap affects activities of daily life. There are 3 possible answers: *yes* gives a score of 4 points, *sometimes* 2, and *no* 0 points. To be sure that none of the components of the DHI play a decisive role, the DHI is distinguishable in a 7-item physical (P) subscale (maximum score 28), a 9-item emotional (E) subscale (maximum score 36), and a 9-item functional (F) subscale (maximum score 36). The DHI is a valid and reliable instrument.^[12]^ It was suggested that a total DHI score of 0 to 30 reflects mild, 31 to 60 moderate, and 61 to 100 severe disability.

A minimal clinical important change (MCIC) of 10% in scores following an intervention is considered to be a clinically significant result.^[13,14]^ However, based on our clinical experience, a change of at least 25% will be more representative of clinical change and probably also less influenced by confounding factors and temporary changes.

### 2.4 Procedure

**Figure 1:**
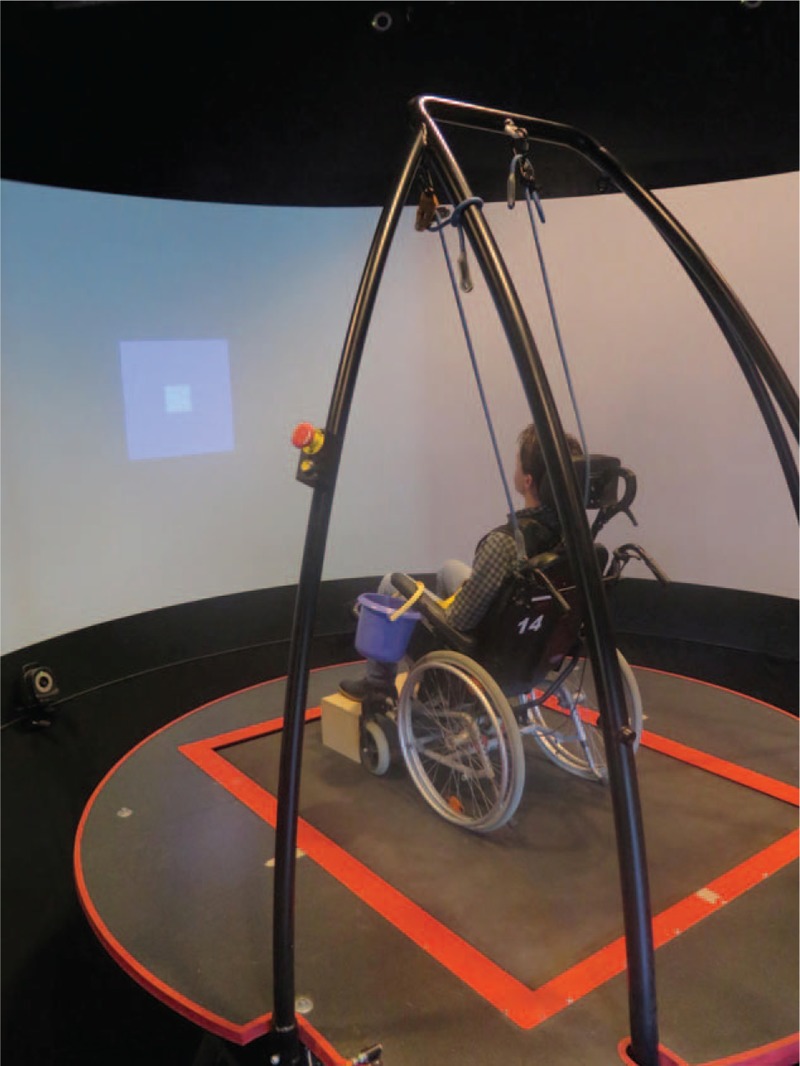
Patient in sitting position on the CAREN platform. CAREN = computer-assisted rehabilitation environment.

The 6 degrees-of-freedom motion base of the (computer-assisted rehabilitation environment) CAREN system (Motek Medical, Amsterdam, the Netherlands) at the MRC in Doorn, the Netherlands was used. A wheelchair with head and backrest was put on the platform and people were seated with their eyes closed in a nonvirtual environment (Fig. 1).

At a frequency of 0.2 Hz, a vertical sinusoidal stimulus was administered, as this frequency has the most nausea-inducing capabilities.^[15]^ Subsequently, we could use the largest possible displacement of the CAREN system without inducing extra passive body and head movements as would be the case in a fore–aft and side-to-side stimulus.

The following displacements were used: 10, 20, 30, and 40 cm. Every minute the Misery score (MISC 1–6) was obtained: MISC 1 = no nausea, MISC 2 = initial symptoms, but no nausea, MISC 3 = mild nausea, MISC 4 = moderate nausea, MISC 5 = severe nausea, and MISC 6 = vomiting. Discontinuation of the stimulus occurred when a MISC 5 (severe nausea) was reached.^[11,16]^

Maximum stimulus duration was 20 minutes. If a person did not reach MISC 4 for a specific stimulus within 20 minutes, the next stimulus day an increased displacement was used. Maximum duration of the desensitization protocol was 12 sessions over a period of 4 weeks with a frequency of 3 sessions per week.

### 2.5 Data analysis

For this observational study, SPSS 22 was used for statistical analysis. Because of the expected limited number of patients, the Shapiro–Wilk test for normality will be applied. If the distribution of the data shows no normality, we planned to use the Wilcoxon signed-rank test with the 25th to 75th percentile to assess the different measurement moments. Because of the repeated measures set-up, the Friedman test was used. *P*-values less than 0.05 were considered significant. Bonferroni correction was applied for multiple testing (critical *P*-value: .025).

This study was approved by the Medical Ethical Committee Brabant no MW2016-01.

## 3 Results

**Figure 2:**
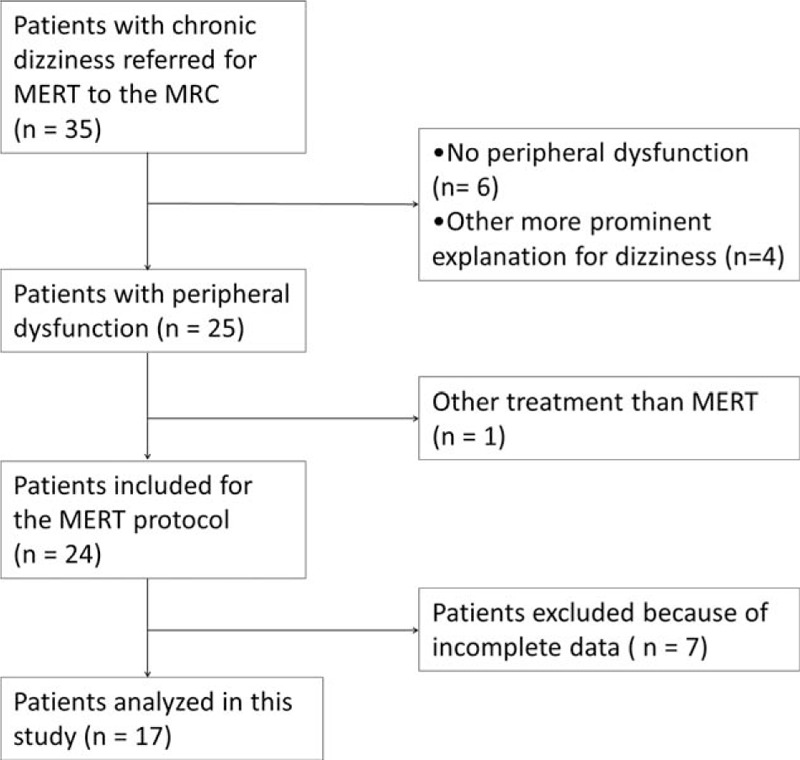
Flow chart of study population.

A flow chart with the process of selecting the cases is presented in Fig. 2. After applying inclusion and exclusion criteria to all 35 consecutive patients who underwent this treatment program, 10 male and 7 female case files were selected for this study. Ten excluded patients had another primary diagnosis (systemic illness, psychiatric disorders, central vestibular disorders, other neurological disorders) explaining their symptoms, 1 patient received other therapy during the program and 7 were unable to fill out the questionnaire. At baseline the median age was 52 years (range 27–60 years) and the median duration of symptoms was 11 months (range 6–192 months). The included patients experienced ongoing vestibular symptoms despite previous treatment, including vestibular rehabilitation (e.g., the Epley maneuver) and medication (e.g., antihistamines or benzodiazepines). The associated disorder causing the peripheral vestibular dysfunction varied among patients. The majority of patients (n = 9) was diagnosed as postvestibular neuritis. There were 2 patients with Ménière's disease, 1 patient with BPPV, and 3 patients with Mal de debarquement. Two other patients without a clear specific peripheral vestibular disorder, however with objective evidence and a history suggestive for peripheral dysfunction, were categorized as nonspecified peripheral vestibular dysfunction.

**Figure 3:**
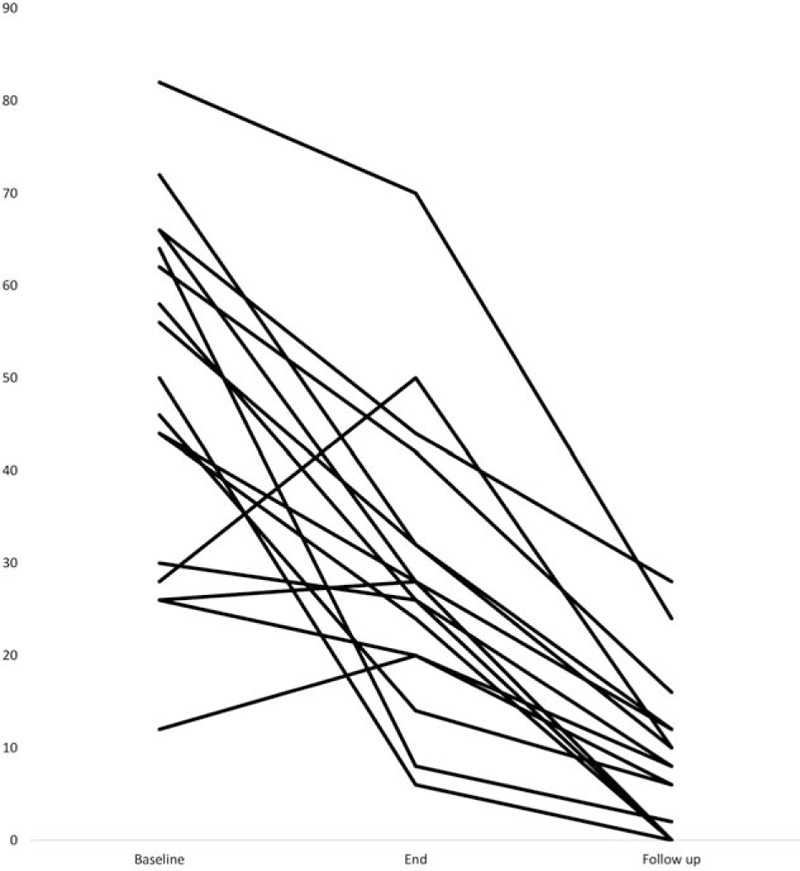
Decent course of the DHI in 17 patients. Fourteen patients with a continued decrease and 3 patients with an increase at the end of treatment and a decrease at follow-up. DHI = Dizziness Handicap Inventory.

The collected data showed no normal distribution, therefore we present these with the median and not the mean values. The median duration of the follow up was 3.8 month (range 2 – 6 month). All 17 patients showed an improvement on the DHI at follow-up; in 14 patients this was already obvious at the end of the rehabilitation period (Fig. 3). In 3 patients, an initial increase was detectable on the DHI; however, at follow- up all 3 patients improved and scored lower than at baseline.

An overview of the DHI (sub)-scores at baseline, end of treatment, and at follow-up is presented in Table 1. The median DHI score at baseline was 50 points, 28.8 points after treatment, and 22 points at follow-up. The Friedman test showed significant differences across multiple test moments *χ*^2^ (2) = 13.556, *P* = <.001. *Post hoc* analysis with Wilcoxon signed-rank tests was conducted with a Bonferroni correction applied, resulting in a significance level set at *P* <.025. The sub-scores of the DHI also showed significant improvement similar to the total DHI score towards the same different measure moments. The improvement between the DHI outcome measurement at the end of the treatment and at follow-up demonstrates no significance.

**Table 1:**
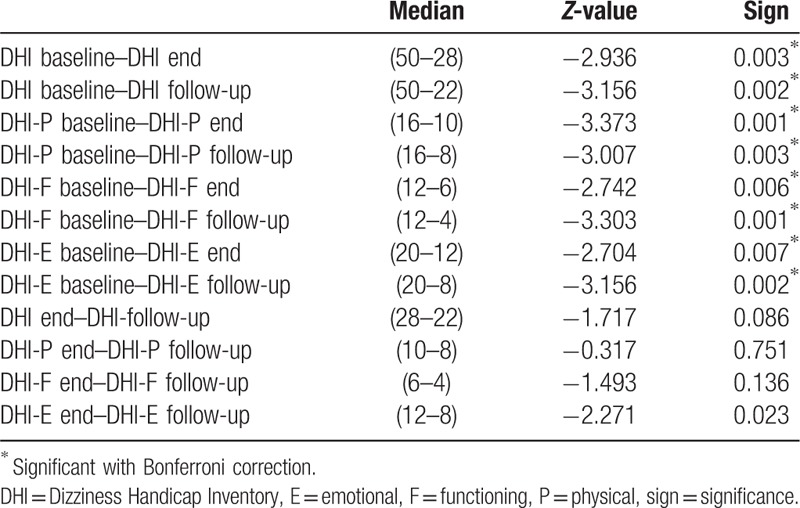
Comparison Dizziness Handicap Inventory and sub-scores E, F, and P at baseline, end, and follow-up.

Thirteen patients achieved at least 25% reduction of symptoms and 3 patients reached at least 10%. One patient did not reach the MCIC of 10%. Four patients had a DHI score of 0 at follow-up.

Temporary exacerbation of vertigo and unsteadiness after a treatment session was commonplace. However, these adverse effects were mild and did not last for more than a few hours. All patients were able complete the MERT treatment protocol.

## 4 Discussion

This study is the first case series that evaluates passive whole body sinusoidal oscillations as a therapy to alleviate symptoms of peripheral vestibular disease in the chronic phase. The results are compelling as they show a large decrease in DHI scores that are sustained at follow-up.

There were no dropouts and only insignificant adverse effects during the therapy sessions. Therefore, although MERT is somewhat uncomfortable, it can be considered a safe and well-tolerated intervention. We hypothesize that the effectiveness is possibly due to the fact that the vertical motion stimulus elicits an otolith stimulation without the commonly expected concomitant stimulation of the semicircular canals. With continued stimulation, this might influence the experienced pitch/roll amplitude and hence dizziness and nausea in a positive way. We hypothesize further that the effect of the MERT treatment is the result of central compensation, causing symptoms of peripheral vestibular dysfunction to disappear or decline. Although originally developed for motion sickness, the subjective vertical mismatch theory may offer an explanation for the central compensatory mechanisms in case of a sensory conflict as is the case in vestibular disorders.^[11]^ In this theory, symptoms can occur when the perceived vertical and expected vertical variate. The theory postulates the idea that by adding a novel sensory input to the system, sensory information can be reweighted. This decreases the subjective vertical mismatch and might consequently diminish symptoms.

Several limitations of this study will be described in the following. Firstly, the sample size was relatively small (n = 17), which makes it difficult to generalize the results. But, we would like to emphasize that this is a pilot study. Also, the patient population is rather heterogeneous. Still, even in this heterogeneous sample large effect sizes are seen. So, it seems worthwhile to conduct a large-scale study based on the results of this study

Secondly, it is axiomatic that there was no control group. In its defense, however, it should be noted that all patients had a prolonged duration of symptoms and already underwent vestibular rehabilitation interventions.

Thirdly, there was a large disparity concerning the age of the participants and therefore we cannot exclude the influence of the aging process in vestibular rehabilitation. However, the contribution of age in our study population seems weak because the complaints about dizziness existed for a relatively long period and decreased in a short time frame. Moreover, symptom relief was achieved in only (a maximum of) 12 sessions and therapeutic effects were maintained at follow-up.

In comparison, vestibular rehabilitation therapy takes much longer and focuses more on self-administration of head exercises.^[4]^ It requires a lot of commitment for patients to perform exercises that, at the start of the therapy, exacerbate symptoms. In our MERT treatment, the patients underwent the therapy passively in a controlled environment with the focus on administering nauseating stimuli with slowly increasing intensity. The increase in symptoms during the sessions was usually quite slow, so patients might feel safer in this environment where they are not overwhelmed by the symptoms.

The MERT protocol is given 3 times per week, habituation studies to simulator sickness point toward a session frequency of 3 times a week for habituation to occur (habituation usually occurs in 6 sessions).^[17]^ Our results are in accordance with these results and we think that a therapy frequency of 3 times a week should be, generally speaking, advisable.

Recent developments in the conservative treatment of symptoms of vestibular disease showed additive effect on a vestibular rehabilitation regime with the use of a roll dome, head-mounted display, optokinetic drum, and a home video.^[18]^ However, all these elements were part of the additive therapeutic regime in that study, and on that account, the specific effect of each of these elements was not clear. Furthermore, their subjects received therapy 2 times a week for 8 weeks. This might be a suboptimal frequency for quick desensitization. One might hypothesize that adding a roll dome and/or optokinetic drum through virtual reality to our procedure can also have an additive effect. Interestingly, virtual reality-assisted therapy for the treatment of vestibular symptoms was used successfully and vestibular symptoms diminished within 4 to 6 weeks after the start of the intervention.^[19–21]^

We conclude that analogous to the military motion sickness desensitization programs, it seems possible to habituate subjects with peripheral vestibular disease to vestibular stimuli of increasing intensity with a theoretically similar, yet less provocative protocol. As noted by others, there is moderate to strong evidence available that vestibular rehabilitation reduces symptoms and improves functioning in daily life, but also that none of the specific vestibular programs is more effective than the other in reducing symptoms.^[4]^ Therefore, both from a cost-effective perspective and also from a participatory view of functioning in daily life, it is worthwhile to switch the research focus to the types of therapy that lead to quicker results. Virtual reality and motion-assisted therapy might be of added value in this respect.

## 5 Conclusion

The results of this pilot study with the MERT rehabilitation for vestibulopathies appear to be compelling with a considerable reduction in the DHI score. Together with the fact that the result is reached in a maximum of 12 sessions in 4 weeks’ time it is a viable treatment option. However, because of the inherent limitations due to case series, the results should be interpreted with caution. In our opinion, the positive outcome makes it worthwhile to conduct a randomized clinical trial for further assessment.
